# Gallbladder Volvulus in an Elderly Female With Severe Kyphoscoliosis

**DOI:** 10.7759/cureus.36256

**Published:** 2023-03-16

**Authors:** Kavina Sidhu, Doruk Seyfi, Michael Crawford, Geoffrey Watson

**Affiliations:** 1 Hepatobiliary and Gastrointestinal Surgery, Royal Prince Alfred Hospital, Sydney, AUS; 2 Tissue Pathology, Royal Prince Alfred Hospital, Sydney, AUS

**Keywords:** laparoscopic cholecystectomy (lc), congenital defect, acute cholecystitis, wandering gallbladder, floating gallbladder, haemorrhagic gangrene, acalculous cholecystitis, gallbladder volvulus, kyphoscoliosis, gallbladder torsion

## Abstract

Gallbladder volvulus is a very rare complication of a congenital defect in gallbladder development also known as a "floating" gallbladder and often presents in the elderly. Proposed aetiologies include loss of abdominal fat and kyphoscoliosis. We present a patient with severe lumbar scoliosis centred on L2, producing a lumbar vertebral distortion of about 30 degrees concave to the right, resulting in right hemiabdomen volume loss. The mechanical interaction between the gallbladder fundus and compressed viscera transmits abnormal ambulatory forces from the distorted right pelvic brim into the abdomen predisposing to gallbladder torsion. Laparoscopic cholecystectomy was performed without complication and the patient had an uneventful recovery.

This case demonstrates the challenges of diagnosing gallbladder torsion preoperatively. A high level of clinical suspicion is vital especially in elderly patients to enable timely surgical intervention to reduce morbidity and mortality.

## Introduction

Gallbladder volvulus is rare and occurs due to torsion of the gallbladder on its mesentery along the axis of the cystic duct and cystic artery [1]. First reported by Wendel in 1898, there are less than 500 documented cases with an incidence of 1:365520 hospital admissions and a 3:1 female predominance [1-4]. An increase in frequency has been noted in the 21st century, likely due to improved investigative tools and increased life expectancy as it is more frequent in elderly patients [4]. Spinal deformities cause anatomical changes, volume loss and abnormal transmission of forces predisposing to unusual pathology such as gallbladder torsion [5].

We discuss the presentation of gallbladder torsion in an elderly female with significant kyphoscoliosis. This case highlights the importance of clinical judgement and emergent surgical intervention as there are no radiological signs to diagnose gallbladder volvulus unequivocally.

## Case presentation

An elderly, cachectic lady in her 80s presented with a three-day history of acute on chronic abdominal pain. She had intermittent generalized pain for the past six weeks. The pain progressively worsened and became more constant and localized in the right upper quadrant with associated nausea, anorexia and fatigue. On examination, she was hypertensive and afebrile with a soft but tender right abdomen. There were no palpable masses.

**Figure 1:**
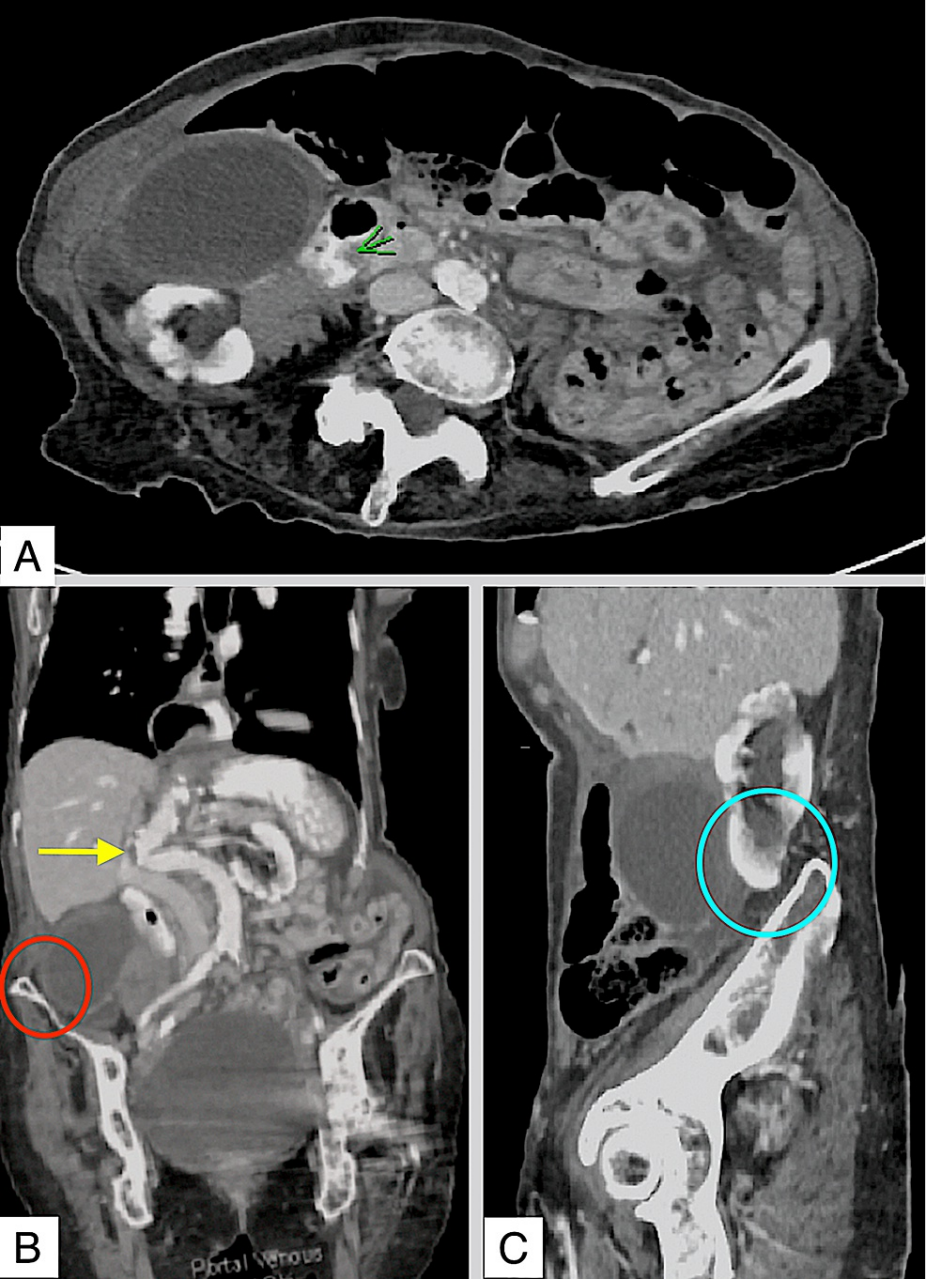
Computed tomography of the abdomen and pelvis with oral and intravenous contrast. (A) Axial computed tomography image demonstrating an ill-defined heterogeneous density within the cystic duct likely an obstructing focus (green arrow). (B) Coronal computed tomography image demonstrating the degree of scoliosis and aortic kinking (yellow arrow) as well as the proximity of the gallbladder fundus to the ilium (red circle). (C) Non-weight bearing sagittal computed tomography image shows the right kidney lying close to the right posterior iliac crest (blue circle).

She had an elevated white cell count of 15.8x10^9^/L (normal range: 4-11x10^9^/L), C-reactive protein of 0.6 mg/L (normal range: <5 mg/L) with normal liver function tests and lipase. There were no clinical or biochemical signs of organ dysfunction or sepsis. Computed tomography demonstrated acute cholecystitis without cholelithiasis and an ill-defined heterogeneous density within the cystic duct likely an obstructing focus (Figure 1A). In retrospect, this obstruction was likely the point of torsion. There was an associated biliary tree and pancreatic duct dilatation as well as a small volume of intra-abdominal free fluid predominantly seen in the paracolic gutters extending to the pelvis. Ultrasound of the abdomen was not performed as there was no service available after hours at the time of presentation.

There was scoliosis of the lumbar spine centred on L2, producing a lumbar vertebral distortion of about 30 degrees with convexity towards the right, grade 1 anterolisthesis at L4-L5 and vertebral height loss at L2 and L3. The non-weight bearing computer tomography images show the anatomical changes caused by significant scoliotic deformity such as aortic kinking, descended position of the gallbladder with the proximity of gallbladder fundus to the ilium (Figure 1B) and the right kidney lying close to the right posterior iliac crest (Figure 1C). Keeping in mind these are non-weight-bearing images, to fully appreciate the anatomical variation brought about by kyphoscoliosis, changes in a weight-bearing state would then need to be conceptualized.

**Figure 2:**
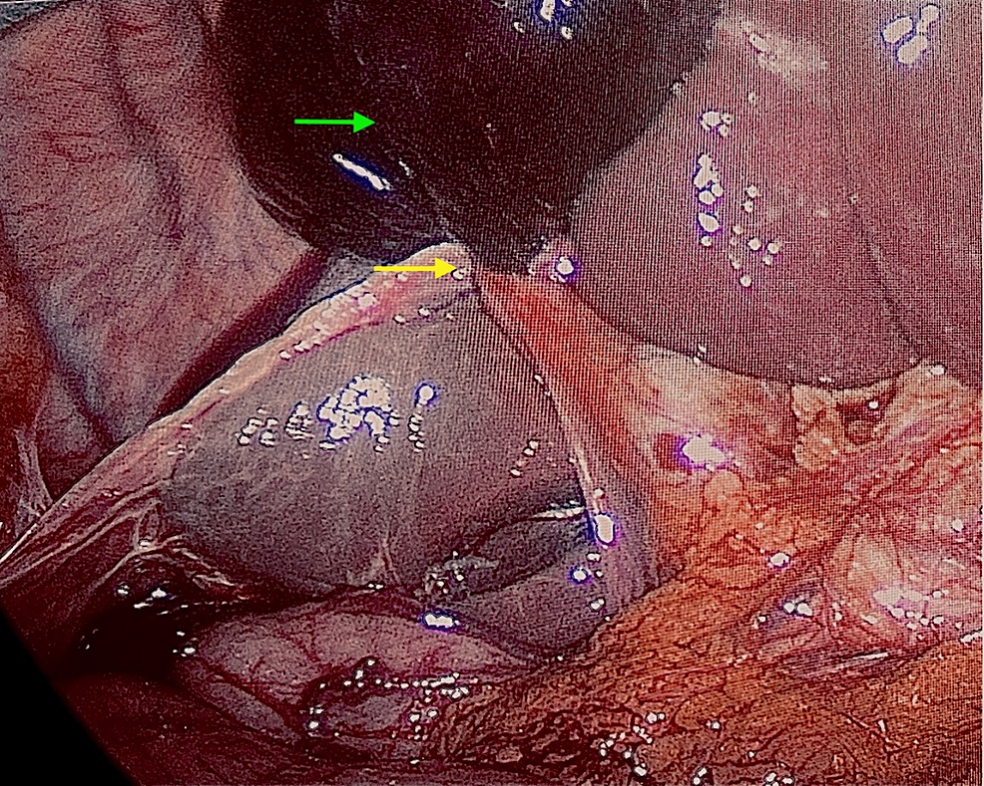
Intraoperative image of gallbladder torsion. Complete volvulus of a distended gallbladder around the cystic duct and artery (yellow arrow) with haemorrhagic gangrene of the gallbladder (green arrow).

**Figure 3:**
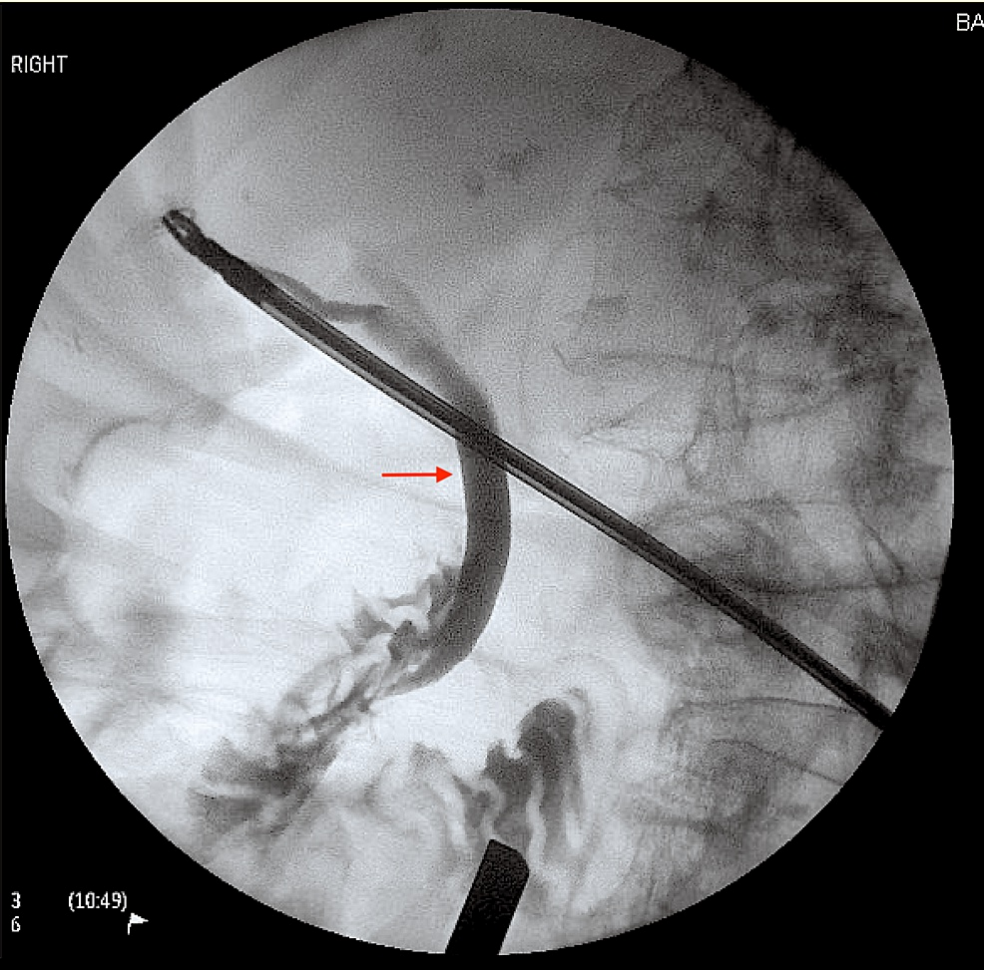
Image of intraoperative cholangiogram. Intraoperative cholangiogram showed dilated common bile duct (red arrow) with good flow into the duodenum and intrahepatic ducts without evidence of filling defects.

She was admitted to the hospital for laparoscopic cholecystectomy and initially managed with intravenous antibiotics, analgesia and venous thromboembolism prophylaxis. Laparoscopic cholecystectomy the following day revealed complete volvulus of a distended gallbladder around the cystic duct and artery with visible macroscopic ischaemia (Figure 2). The gallbladder was decompressed via the right mid-clavicular port site and detorted. The cystic artery was semi-necrosed due to ischaemia. Blood supply to the distal cystic duct was unaffected, with normal flow of bile. An intraoperative cholangiogram showed dilated common bile duct with good flow into the duodenum and intrahepatic ducts without evidence of filling defects (Figure 3).

**Figure 4:**
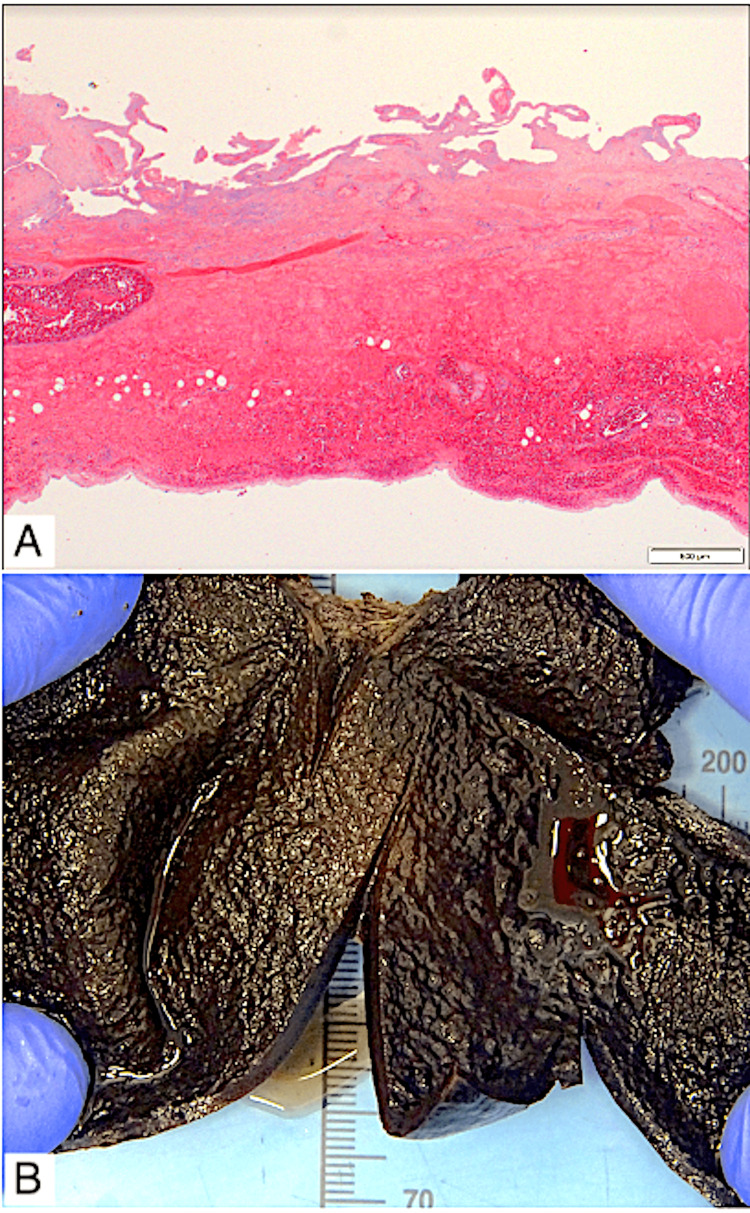
Histopathology images of the gallbladder. (A) Microscopic pathology of the gallbladder wall showing marked venous engorgement and diffuse haemorrhage. The wall had undergone necrosis, and the mucosal epithelium sloughed. No active inflammation. (B) Macroscopic pathology of the formalin-fixed gallbladder, showing diffuse mural haemorrhage and oedematous mucosal pattern.

Laparoscopic cholecystectomy was achieved without complication and the patient had an uneventful recovery. Histopathology confirmed gallbladder volvulus with engorged veins and full-thickness wall haemorrhage and necrosis (haemorrhagic gangrene) (Figure 4). No calculi were present.

## Discussion

Congenital variation in peritoneal attachment results in the five recognized positions of the gallbladder in relation to the liver: (1) completely embedded in the liver (intrahepatic), (2) closely attached to the inferior liver by peritoneum, (3) a complete mesentery but held close to the liver, (4) a complete long mesentery and (5) an incomplete mesentery attached along the cystic duct. Only types 4 and 5 predispose to volvulus as the gallbladder hangs free, floating in the abdominal cavity and allowing it to twist around the cystic pedicle [1,5,6]. This mechanical twist results in bile flow interruption, impaired venous outflow and increased surface tension on the gallbladder with subsequent compromised arterial perfusion leading to ischaemia, necrosis followed by perforation and bilious peritonitis which increases the mortality rate to 5% if not diagnosed promptly [1-5].

Mechanical events such as sudden changes in body position, peristalsis of adjacent viscera and blunt trauma may be inciting events [1,3,5]. Hormonal change such as increased cholecystokinin production after a fatty meal leads to gallbladder peristalsis facilitating torsion [1,5]. Predisposing factors such as loss of visceral fat and elasticity with aging and liver atrophy result in an elongated gallbladder mesentery [1,5]. Weight loss and bariatric surgery are also contributing factors [1,3]. Spinal deformities such as kyphosis and scoliosis result in a lower position of the gallbladder [5]. Our patient had severe lumbar scoliosis causing volume loss in the right hemiabdomen. The gallbladder fundus lies in close relation to the iliac crest permitting mechanical interaction during weight bearing predisposing to torsion.

Gallbladder volvulus is commonly misdiagnosed as acute cholecystitis. Definitive diagnosis is usually achieved intraoperatively and only 10% of cases are diagnosed preoperatively [1,3,4,7]. Differentiating factors from acute cholecystitis include low frequency of fever, poor response to antibiotics and acute onset abdominal pain [1,5,8]. In the literature, only four cases have been diagnosed with pre-operative imaging [1,5].

Clinical features include right upper quadrant pain, guarding and palpable painful gallbladder. Complete rotation (>180 degrees) presents with acute severe right upper quadrant pain and vomiting versus incomplete torsion (<180 degrees) with intermittent symptoms mimicking biliary colic [1,3,6]. Lau et al. described three triads suggestive of gallbladder torsion [1,4,5,7]: (1) patient’s characteristics (thin elderly patients, with chronic lung disease, or spinal deformities),​​​​​​ (2) symptoms (acute, abdominal pain, with early onset vomiting) and (3) signs (abdominal mass, absence of sepsis or jaundice, discrepancy in pulse and temperature).

The majority of cases (70-80%) are not associated with cholelithiasis. One study of 245 patients found cholelithiasis in 24.4% of patients [1,3,5,8]. Ultrasound usually reveals an image similar to acute cholecystitis (large, thickened gallbladder in a transverse plane outside of fossa with pericholecystic fluid) but may be extremely specific in diagnosing volvulus if the gallbladder is located outside of the fossa and inferior to the liver described as a wandering or pedunculated gallbladder [1,3,5]. Colour doppler flow could be used to visualize arterial flow [1,8]. A cystic duct knot is an echogenic nodule at the neck of the gallbladder which represents a twisted cystic duct allowing early diagnosis. Computed tomography is nonspecific but demonstrates a distended, horizontally displaced, thickened, hypoattenuating, poorly enhanced gallbladder wall and a whirl sign (twisted cystic pedicle) which are pathognomonic for gallbladder volvulus [1-3,7].

Once diagnosed, emergent detorsion and cholecystectomy should be performed to prevent perforation and biliary peritonitis for an excellent prognosis [1,5]. In contrast to acute cholecystitis, it is unlikely to respond to conservative treatment or percutaneous cholecystostomy [1-3]. A laparoscopic approach is preferred as decompression, and detorsion of the gallbladder reduces the risk of bile duct injury [1,5,6].

## Conclusions

This case demonstrates the challenges of diagnosing gallbladder torsion preoperatively as there are no radiological signs to unequivocally diagnose gallbladder volvulus. It should be considered in patients with clinical and radiological features of acalculous cholecystitis with a gallbladder in an unusual location. Kyphoscoliotic deformity produces a secondary reduction in intra-abdominal volume and abnormal transmission of forces which may predispose to unusual pathologies. A high level of clinical suspicion is necessary to diagnose gallbladder volvulus, especially in elderly patients to enable timely surgical intervention to reduce morbidity and mortality.
